# Further Delineation of CANT1 Phenotypic Spectrum and Demonstration of Its Role in Proteoglycan Synthesis

**DOI:** 10.1002/humu.22104

**Published:** 2012-04-26

**Authors:** Mathilde Nizon, Céline Huber, Fabio De Leonardis, Rodolphe Merrina, Antonella Forlino, Mélanie Fradin, Beyhan Tuysuz, Bassam Y Abu-Libdeh, Yasemin Alanay, Beate Albrecht, Lihadh Al-Gazali, Sarenur Yilmaz Basaran, Jill Clayton-Smith, Julie Désir, Harinder Gill, Marie T Greally, Erkan Koparir, Merel C van Maarle, Sara MacKay, Geert Mortier, Jenny Morton, David Sillence, Catheline Vilain, Ian Young, Klaus Zerres, Martine Le Merrer, Arnold Munnich, Carine Le Goff, Antonio Rossi, Valérie Cormier-Daire

**Affiliations:** 1 Departement de Génétique, INSERM U781, Université Paris Descartes-Sorbonne Paris Cité, Institut Imagine, Hôpital Necker-Enfants Malades (AP-HP)Paris, France; 2 Department of Molecular Medicine, University of PaviaPavia, Italy; 3 Division of Genetics, Department of Pediatrics, Cerrahpasa Medical Faculty, Istanbul UniversityIstanbul, Turkey; 4 Pediatrics and Genetics, Makassed Hospital, Jerusalem, Al-Quds Medical SchoolJerusalem; 5 Pediatric Genetics, Department of Pediatrics, Acibadem University School of MedicineIstanbul, Turkey; 6 Institute for Human Genetics, University of HufelandstrEssen, Germany; 7 Department of Paediatrics, Faculty of Medicine and Health Sciences, United Arab Emirates UniversityAl-Ain, United Arab Emirates; 8 Department of Medical Genetics, Cerrahpasa Medical Faculty, Istanbul UniversityIstanbul, Turkey; 9 Genetic Medicine, Manchester Academic Health Science Centre, University of Manchester; Central Manchester University Hospitals NHS Foundation Trust, St Mary's HospitalManchester, United Kingdom; 10 Department of Medical Genetics, Hôpital Erasme-ULBBrussels, Belgium; 11 National Centre for Medical Genetics, Our Lady's Children's HospitalCrumlin, Dublin, Ireland; 12 Department of Clinical Genetics, Academic Medical CentreAmsterdam, The Netherlands; 13 Provincial Medical Genetics Program, Eastern HealthSt. John's, Newfoundland, Canada; 14 Center for Medical Genetics, Antwerp University Hospital and University of AntwerpAntwerp, Belgium; 15 Clinical Genetics Unit, Birmingham Women's HospitalBirmingham, United Kingdom; 16 Department of Genetic Medicine, University of SydneyNew South Wales, Australia; 17 Department of Clinical Genetics, Leicester Royal InfirmaryLeicester, United Kingdom; 18 Department of Human Genetics, Aachen UniversityAachen, Germany

**Keywords:** Desbuquois dysplasia type 1 and type 2, *CANT1*, *CHST3*, proteoglycan metabolism

## Abstract

Desbuquois dysplasia (DD) is characterized by antenatal and postnatal short stature, multiple dislocations, and advanced carpal ossification. Two forms have been distinguished on the basis of the presence (type 1) or the absence (type 2) of characteristic hand anomalies. We have identified mutations in calcium activated nucleotidase 1 gene (*CANT1)* in DD type 1. Recently, *CANT1* mutations have been reported in the Kim variant of DD, characterized by short metacarpals and elongated phalanges. DD has overlapping features with spondyloepiphyseal dysplasia with congenital joint dislocations (SDCD) due to Carbohydrate (chondroitin 6) Sulfotransferase 3 (*CHST3*) mutations. We screened *CANT1* and *CHST3* in 38 DD cases (6 type 1 patients, 1 Kim variant, and 31 type 2 patients) and found *CANT1* mutations in all DD type 1 cases, the Kim variant and in one atypical DD type 2 expanding the clinical spectrum of hand anomalies observed with *CANT1* mutations. We also identified in one DD type 2 case *CHST3* mutation supporting the phenotype overlap with SDCD. To further define function of *CANT1*, we studied proteoglycan synthesis in *CANT1* mutated patient fibroblasts, and found significant reduced GAG synthesis in presence of β-D-xyloside, suggesting that *CANT1* plays a role in proteoglycan metabolism. Hum Mutat 33:1261–1266, 2012. © 2012 Wiley Periodicals, Inc.

## Introduction

Desbuquois dysplasia [DD; MIM# 251450] is a severe autosomal recessive chondrodysplasia belonging to the multiple dislocations group (group 20 in the International Classification of Bone Disorders) [Warman et al., 2011]. First described by Desbuquois et al. in 1966 [Desbuquois and Rossignol, 1966], it is characterized by prenatal and postnatal short stature (−4 SD to −10 SD), joint laxity, multiple dislocations, brachydactyly, and facial dysmorphism (prominent eyes and flat face). Some radiological features are mandatory for the diagnosis including an advanced carpal and tarsal bone age, and short long bones with “Swedish key” appearance of the proximal femur. Additional anomalies include hydramnios, cardial septal defect, lung hypoplasia, glaucoma and mental retardation. At the adult age, final stature is about 114 cm (−8.5 DS) and orthopedic complications often limit the ambulation [Le Merrer et al., 1991].

We have previously described clinical and genetic heterogeneity in DD. Based on the presence of hand anomalies, namely, accessory ossification center distal to the second metacarpal, bifid distal phalanx, or delta phalanx of the thumb, DD type 1 has been defined while DD type 2 was distinct by the absence of hand anomalies [Faivre et al., 2004]. Recently, Kim et al. have distinguished a variant that is characterized by very short metacarpals and elongated phalanges without accessory ossification center [Kim et al., 2010].

Up till now, *CANT1* (calcium activated nucleotidase 1) mutations have been reported in Desbuquois dysplasia type 1 and Kim variants [Faden et al., 2010; Furuichi et al., 2011; Huber et al., 2009; Laccone et al., 2011]. More recently, *CHST3* (carbohydrate (chondroitin 6) sulfotransferase 3) mutations, involved in spondyloepiphyseal dysplasia with congenital joint dislocations [SDCD; MIM# 143095], which shares some features with DD including multiple dislocations and joint hyperlaxity, have been reported in one case of DD type 2 [Unger et al., 2010]. Furthermore, many clinical features are common to spondyloepiphyseal dysplasia, Omani type, or humerospinal dysostosis other well-described entities caused by defects in *CHST3* [Hermanns et al., 2008; Thiele et al, 2004; Van Roij et al., 2008].

The aim of our study was to screen *CANT1* and *CHST3* in 38 cases of Desbuquois dysplasia. The function of *CANT1* is unknown. However, considering the clinical overlap between DD and *CHST3* conditions characterized by undersulfation of glycosaminoglycan (GAG) chains, we hypothesized that *CANT1* may be also involved in proteoglycan synthesis and performed biochemical analysis to further define its role.

## Materials and Methods

### Patient Recruitment and Clinical Assessment

Thirty-eight patients with DD have been included in this study. They were recruited through either the French reference center for skeletal dysplasias or international collaborations. All patients fulfilled the diagnosis criteria for DD, namely, pre- and postnatal growth retardation, joint laxity, short long bones, advanced bone age and “Swedish key” appearance of the proximal femur. Among them, six patients were classified as DD type 1, based on the presence of at least one of the following hand features: (1) an accessory ossification center, (2) a delta phalanx of the thumb, or (3) a bifid distal phalanx of the thumb. One patient fulfilled the diagnosis criteria for Kim variant. Thirty-one patients were classified as DD type 2 although one of them presented some atypical hand anomalies.

The study was approved by our hospital ethics board. Written informed patient and parent consents were obtained for additional genetic investigations.

### DNA Analysis

Linkage analysis at *CANT1* and *CHST3* loci was first performed in consanguineous families. Mutation screening was then performed by direct sequencing of the exons and the exon–intron boundaries of *CANT1* and *CHST3* for compatible consanguineous and nonconsanguineous families.

Primer sequences are summarized in supporting data (Supp. Tables S1 and S2). Sequences were aligned with the known *CANT1* (NCBI reference sequence: NG_016645.1) and *CHST3* (NCBI reference sequence: NG_012635.1) coding sequences. Nucleotide numbering reflects cDNA numbering with +1 corresponding to the A of the ATG translation initiation codon in the reference sequence, according to journal guidelines (www.hgvs.org/mutnomen). The initiation codon is codon 1.

All variants identified in this study have been submitted to Leiden Open Variation Database (http://www.lovd.nl/CANT1).

The Alamut software was used to study retained mutation sites among different species. The possible functional impact of amino acid changes was predicted by the PolyPhen-2 program (Polymorphism Phenotyping v2, http://genetics.bwh.harvard.edu/pph2) [Adzhubei et al., 2010] and SIFT (Sorting Intolerant from Tolerant).

### RNA Analysis

Total RNA was extracted from peripheral blood leucocytes of patient 8 and of control patients by a standard method. The RNA samples were reverse transcribed with a RT-PCR kit. Primers used for PCR of *CANT1* cDNA were *5′-GCTATCCGACCTGATTGTTTTC-3′* and *5′-GTTCACCACATCACCCGTAGT-3′*. The RT-PCR products were separated on a 1.5% agarose gel.

### Metabolic Labeling of Fibroblast Cultures

Skin fibroblasts from patients and controls were cultured in minimum essential medium (MEM) with 10% fetal calf serum and antibiotics at 37°C in a humidified atmosphere containing 5% CO_2_.

Proteoglycan labeling experiments were performed in triplicate; confluent cells in 10 cm petri dishes were preincubated for 4 hr with or without 1 mM p-nitrophenyl β-d-xylopiranoside in MEM containing 250 µM cold Na_2_SO_4_ without FCS in 5% CO_2_ at 37°C. Cells were then double labeled with 150 µCi/ml Na_2_[^35^S]O_4_ and 40 µCi/ml [6-^3^H]glucosamine in the same medium for 24 hr as described previously [Rossi, et al., 1998]. At the end of the labeling period an equal volume of 100 mM sodium acetate buffer, pH 5.8, containing 8 M urea, 4% Triton X-100, 20 mM ethylenediaminetetraacetic acid, 20 mM N-ethylmaleimide (NEM), and 1 mM phenylmethanesulfonyl fluoride (PMSF) was added to the medium. The cell layer was harvested in 50 mM sodium acetate buffer, pH 5.8, containing 2 M urea, 2% Triton X-100, and an aliquot was used for protein content determination with the bicinchoninic acid (BCA) Protein Assay (Pierce) while the remainder was added to the medium. Samples were loaded on 1 ml diethylaminoethyl (DEAE) Sephacel columns; after columns washing with 50 mM sodium acetate buffer, pH 6.0, 8 M urea, 0.15 M NaCl, 0.5% Triton X-100 and proteinase inhibitors, proteoglycans, and hyaluronic acid were eluted with 1 M NaCl in the same buffer, recovered by precipitation with 9 volumes of ethanol and desalted by ultrafiltration with Centricon-10. Proteoglycans were then separated from hyaluronic acid by digestion with 3 units of *Streptomyces* hyaluronidase (Seikagaku) in 20 mM sodium acetate, pH 6.0, 75 mM NaCl at 60°C overnight followed by ultrafiltration with Centricon-10. Proteoglycans in the retentate were quantified by ^35^S activity counting and normalized to the protein content; hyaluronic acid in the filtrate was measured by ^3^H activity and normalized to the protein content.

### Size Exclusion Chromatography of GAG Chains

Labeled proteoglycans synthesized by cells in absence of p-nitrophenyl β-D-xylopiranoside and purified as described above, were β-eliminated to release GAG chains by alkaline digestion with 0.125 M NaOH followed by reduction with 1 M NaBH_4_ overnight at room temperature. After neutralization with acetic acid, samples were lyophilized, dissolved in 4 M guanidinium chloride, 50 mM sodium acetate buffer, pH 6.0, 0.5% Triton X-100 and loaded on a Superose 6 10/300GL column (GE) eluted in the same buffer. ^35^S activity was measured by scintillation counting in collected fractions.

## Results

We identified eight distinct *CANT1* mutations, including five novel mutations in six DD type 1 cases, one Kim variant, and one DD type 2 case with atypical hand anomalies (Table 1). Among them, three were missense mutations (p.Arg300His, p.Ile374Asn and p.Ser303Arg), four nonsense mutations (p.Tyr178Leufs*3, p.Ala34Phefs*56, p.Leu93Valfs*89, and p.Gln120Lysfs*10) and one intronic splice site mutation (c.-342+1G>A). All mutations cosegregated with the disease and were not identified in 200 control chromosomes. The missense mutations were located in the region encoding the apyrase domain within a highly conserved region and were predicted as damaging using Polyphen and Alamut softwares. The intronic mutation was predicted to alter a donor site in 5′UTR according to the Human Splicing Finder predicting splicing software [Desmet et al., 2009]. To analyze the effect of this mutation, we performed *CANT1* cDNA analysis on patient 8 RNA extracted from leucocytes and found no product by RT-PCR supporting an absence of *CANT1* mRNA transcription due 5′UTR splice site alteration (data not shown).

**Table 1:** *CANT1* Mutations in Desbuquois Dysplasia

No.	Origin	Consanguinity	Birth length	Growth retardation	Joint dislocation	Hand anomalies	Other anomalies	*CANT1* mutations and AA changes
1	Israel	Yes	NI	+	+	Yes	−	Father c.899G>A (p.Arg300His) Mother c.899G>A (p.Arg300His)
2	Morocco	Yes	41 cm	+	Hip, knee, shoulder	Yes	Retrognathia, glaucoma	Father c.1121T>A (p.Ile374Asn) Mother c.1121T>A (p.Ile374Asn)
3	The Netherlands (Surinamese Hindustan descent)	No	5ePer (TOP at 21 SA)	+	Hip, elbow	Metacarpal II hypoplasia, extra ossification center distal to the second metacarpal, duplicated distal phalanx of the thumb	↑ nuchal translucency, coronal clefting, sacral agenesia, large big toe, toe syndactyly, equinovarus feet	Father c.100delinsTT (p.Ala34Phefs^*^56) Mother c.358delC (p.Gln120Lysfs^*^10)
4	Turkey	Yes	NI	+	Knee	Extra ossification center distal to the second and third metacarpal	Proptotic eyes, blue sclerae, flat face	Father c.531_532delCT (p.Tyr178Leufs^*^4) Mother c.531_532delCT (p.Tyr178Leufs^*^4)
5	Yemen	Yes	NI	+	Hip	Bifid distal phalanx of the thumb, finger dislocations	Club feet, narrow thorax, simian crease, patent foramen ovale, patent ductus arteriosus, craniosynostosis	Father c.531_532delCT (p.Tyr178Leufs^*^4) Mother c.531_532delCT (p.Tyr178Leufs^*^4)
6	Bangladesh	No	31 cm (TOP at 35 SA)	+	Hip, knee, elbow	Extra ossification center distal to the first and the fourth metacarpals, finger dislocations	Hydramnios, ventricular septal defect, coronal vertebral clefts	Father c.277_278delCT (p.Leu93Valfs^*^89) Mother c.100delinsTT (p.Ala34Phefs^*^56)
7	Turkey	Yes	NI	+	Hip, knee	Short metacarpals, elongated phalanges	Elbow limitation, mitral valve prolapse	Father c.909C>G (p.Ser303Arg) Mother c.909C>G (p.Ser303Arg)
8	Turkey	Yes	NI	+	Hip, knee	Thumb digitalization, finger dislocations	Equinovarus feet	Father c.−286+1G>A Mother c. −286+1G>A

The *CANT1* sequences were compared to the reference sequence of *CANT1* (NCBI reference sequence: NG_016645.1) with nucleotide numbering starting at the first adenine of the translation initiation codon ATG.

TOP, termination of pregnancy; NI, no indication.

**Figure 1:**
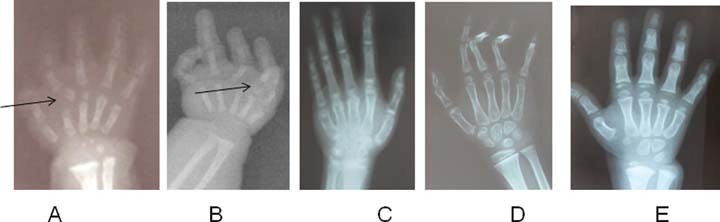
Hand X-rays of Desbuquois dysplasia. **A:** Patient 4 (p.Tyr178Leufs*4) had extra ossification centers distal to second and third metacarpals (2 months old). **B:** Patient 6 (p.Ala34Phefs*56 and p.Leu93Valfs*89) had extra ossification centers distal to the first and the fourth metacarpals associated with finger dislocations (fetal stage). **C:** patient 7 (p.Ser303Arg) presented similar hand anomalies compared to Kim variant with very short metacarpals and elongated phalanges (7 years old). **D:** patient 8 (c.−342+1G>A) showed finger dislocations, epiphyseal interphalangeal anomalies, and thumb digitalization without extra ossification center (2.5 years old). **E:** Desbuquois dysplasia type 2 patient (2 years old). Normal hand (4 years old). Advanced bone age can be observed in all patients.

All mutated patients presented variable range of hand anomalies (Table 1 and Fig. 1). Patients 1–6 had typical hand anomalies. Patient 7 presented with characteristic features of the Kim variant with very short metacarpals and elongated phalanges without extra ossification center. Finally, atypical hand anomalies were noticed in patient 8 including thumb digitalization and severe finger dislocations with epiphyseal anomalies (Fig. 1).

No mutation was found in *CANT1* in the other 30 DD type 2 cases with no hand anomaly apart from advanced carpal ossification center (Fig. 1). However, we identified a missense *CHST3* mutation (p.Leu259Pro), present at the homozygote state in one case (Table 2). This mutation was located in the region encoding the carbohydrate sulfotransferase domain within a highly conserved region, was not identified in 200 control chromosomes and was predicted to be damaging by Alamut software.

**Table 2:** *CHST3* Mutation in DD Type 2

N°	Origin	Consanguinity	Growth retardation	Joint dislocation	Hand anomalies	Other anomalies		*CHST3* mutations
9	Syria	Yes	−7SD	Hip, knee	No	Club feet, elbow limitation, camptodactyly	Father	c.776T>C (p.Leu259Pro)
							Mother	c.776T>C (p.Leu259Pro)

SD, standard deviation.

This patient was first classified as DD based on the presence of knee and hip dislocations and slightly advanced carpal bone age. He also presented a scoliosis, club feet, limited extension in both elbows and camptodactyly of the fifth fingers. At 14 years old, height was 114 cm (−8 DS). He had considerable walking difficulties and, unless numerous surgeries, was in a wheelchair at 11. No mental retardation was noticed.

**Figure 2:**
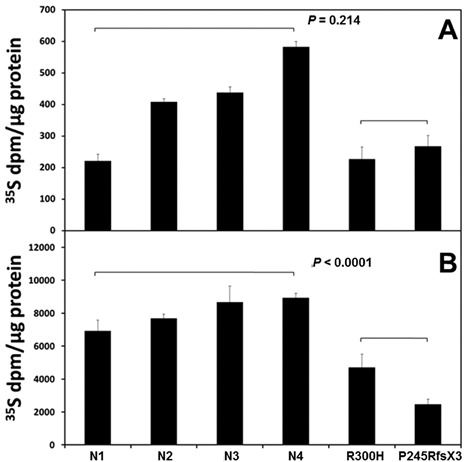
Proteoglycan synthesis in cells incubated in basal medium (**A**) or in presence of β-D-xyloside (**B**). Fibroblasts from Desbuquois dysplasia type 1 patients homozygous for the c.899G>A (p.R300H) and c.734delC (p.P245RfsX3) mutations in CANT1 and four controls (N1-4) were double labeled with [^35^S]sulfate and [^3^H]glucosamine. (**A**) When cells were incubated in basal medium proteoglycan synthesis varied greatly among cultures and patient cells were within normal values. (**B**) A significant reduction of proteoglycan synthesis was observed in patient fibroblasts in presence of β-D-xyloside, a compound that increases glycosaminoglycan synthesis. The ^35^S/^3^H ratio was within normal values (data not shown). Two independent experiments were performed and each experiment was run in triplicate; results are expressed as means ±SD. The statistical significance was calculated with the Student's *t*-test.

Considering the clinical overlap between conditions due to *CHST3* mutations and DD, we finally questioned whether *CANT1* may play a role in proteoglycan and hyaluronic acid metabolism. For this purpose, we metabolically labeled with ^3^H-glucosamine and ^35^SO_4_^2−^ fibroblasts from two previously published DD type 1 patients with *CANT1* homozygous mutations (c.899G>A (p.Arg300His) and c.734delC (p.Pro245Argfs*4) [Huber et al., 2009]) and four age matched controls. No significant difference in proteoglycan synthesis was observed when cells were incubated in basal medium due to huge variability among controls (*P* = 0.214) (Fig. 2A); however, in the presence of β-d-xyloside, a compound which enhances synthesis and secretion of chondroitin and dermatan sulfate chains acting as a chain initiator [Sobue et al., 1987], GAG synthesis in patient cells was significantly reduced compared to control cell lines (*P* < 0.0001) (Fig. 2B).

**Figure 3:**
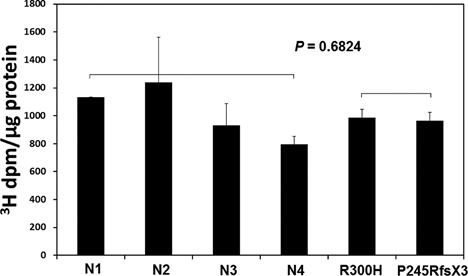
Hyaluronic acid synthesis in cells incubated as in Figure 2. *CANT1* mutations do not affect hyaluronic acid synthesis in the two DD patients. Two independent experiments were performed and each experiment was run in triplicate; results are expressed as means ±SD. The statistical significance was calculated with the Student's *t*-test.

**Figure 4:**
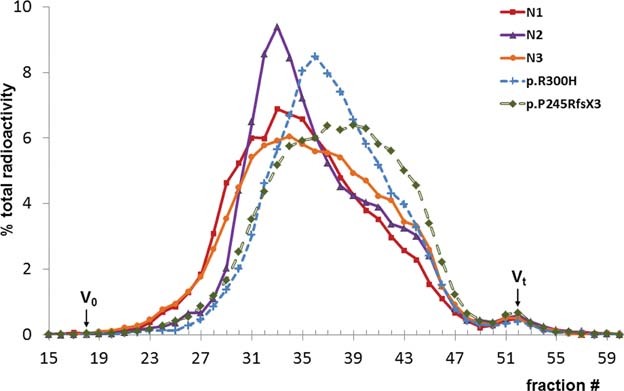
Molecular mass analysis of glycosaminoglycan chains. Glycosaminoglycan chains showed a significant lower mass in patient cells (R300H, P245Rfs*3) compared to the controls (N1-3) as demonstrated by the *K*_av_ values of the peak apex (*K*_av_ = 0.53/0.55 and *K*_av_ = 0.44/0.47, respectively; *P* < 0.01) indicating that oligosaccharide chains are shorter in patient fibroblasts compared to control cell lines.

As an indirect measure of proteoglycan sulfation the ^35^S/^3^H ratio was determined in newly synthesized proteoteoglycans from fibroblast cell lines; the ratio was normal indicating that, within the limits of the technique, CANT1 does not affect proteoglycan sulfation (data not shown). Hyaluronic acid synthesis which occurs in the plasma membrane, a different compartment from proteoglycans, was also within normal values (*P* = 0.682) (Fig. 3). The defect in proteoglycan metabolism was further confirmed by gel filtration chromatography on Superose 6 of the GAG chains released from newly synthesized proteoglycans after β-elimination. GAG chains in the patient cells showed a lower molecular mass compared to the controls (*K*_av_ = 0.53-0.55 and *K*_av_ = 0.44-0.47, respectively; *P* < 0.01) (Fig. 4).

## Discussion

We report here the molecular analysis of *CANT1* and *CHST3* in 38 DD cases. As previously reported, we identified *CANT1* mutations in all DD type 1. We also identified *CANT1* mutation in one Kim variant and in one DD case without any accessory center, but with major finger dislocations, epiphyseal interphalangeal anomalies, and thumb digitalization, expanding the phenotypic spectrum of hand anomalies observed with *CANT1* mutations.

Up till now, 28 patients have been reported with *CANT1* mutations (literature and our present series [Faden et al., 2010; Furuichi et al., 2011; Huber et al., 2009; Laccone et al., 2011]). Among them, 20 patients had characteristic hand anomalies, namely, extra ossification centers or bifid distal phalanx of the thumb with or without additional anomalies such as hypoplasic or low inserted triphalangeal thumbs and phalangeal dislocations. Apart from our case with an homozygous *CANT1* splice site mutation and atypical hand anomalies (thumb digitalization and major phalangeal dislocations but without extra ossification center), only one case has been reported so far with “normal” hands, but no clinical or radiological data were presented [Furuichi et al., 2011]. Finally, six patients were classified as Kim variants characterized by recognizable hand anomaly, including five patients from Japan and Korea and one Turkish patient currently reported [Kim et al., 2010]. While Kim variant was first described in Japan/Korean population sharing a common haplotype and supporting a founder effect in this population, our findings support the existence of Kim variant in other population.

Twenty-four distinct *CANT1* mutations have been reported so far located throughout the gene and including eleven nonsense mutations, eleven missense mutations, one large deletion in 5′UTR and one splice site mutation (current publication [Faden et al., 2010; Furuichi et al., 2011; Huber et al., 2009; Laccone et al., 2011]). The arginine 300 substitution has been identified in 6/28 unrelated DD type 1 patients (p.Arg300Cys [3/6], p.Arg300His [(3/6]). The valine 226 substitution has been identified in five patients with a Kim variant phenotype from Japan/Korea. No other obvious correlation between genotype and phenotype could be established.

DD type 2 patients represented two-thirds of our cohort. All were characterized by normal hands, apart from advanced carpal ossification. *CHST3* mutations have been reported in spondyloepiphyseal dysplasia with congenital joint dislocations and in one case of DD type 2 [Hermanns et al., 2008]. Our DD type 2 case harbored an homozygous p.Leu259Pro mutation previously reported in spondyloepiphyseal dysplasia with congenital joint dislocations. However, some clinical features were atypical for DD, such as camptodactyly and elbow limitation, and were more suggestive of the clinical spectrum reported in CHST3 conditions.

The clinical overlap observed in DD and DTDST/CHST3 conditions, which are due to generalized undersulfation and a lack of 6-O-sulfation of the GAG chains, respectively [Hermanns et al., 2008; Rossi et al., 1998], support the involvement of CANT1 in GAG biosynthesis. Proteoglycan synthesis was within normal value in basal medium; however, in this culture condition, comparisons are difficult and it has been previously reported that proteoglycan synthesis varies greatly among cultures depending on tissue source of the cells, the in vitro age of the culture and as yet unidentified factors [Harper et al., 1987]. It is well known that β-d-xyloside acts as an artificial chain initiator in the Golgi and maximally stimulates chondroitin/dermatan sulfate synthesis, relieving the rate limitation normally exerted by core-protein supply [Robinson et al., 1975]. Thus, by treating cultures with β-d-xyloside we tested the cell ability to synthesize GAGs under condition of markedly stimulated GAG synthesis and we clearly found a significant reduction of proteoglycan synthesis in DD fibroblasts in the presence of β-d-xyloside.

The same defect enhancement has been observed in other disorders of proteoglycan metabolism, namely, the DTDST family of disorders and *CHST3* disorders resulting in generalized undersulfation and a lack of 6-O-sulfation of the GAG chains, respectively. Indeed, the sulfation defect was enhanced when fibroblasts were incubated with xyloside suggesting that this condition might mimic the cartilage situation because it is thought that chondrocytes synthesize higher amounts of proteoglycans than any other tissue.

**Figure 5:**
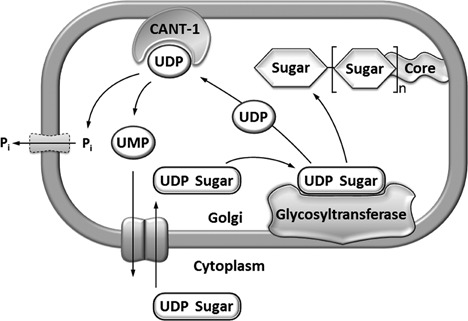
Schematic representation of the suggested role of *CANT1* in proteoglycan metabolism. UDP sugars are transported into the lumen of the Golgi apparatus where sugars are transferred by specific glycosyltransferases to the growing GAG chains. UDP, the other reaction product, is hydrolysed to UMP and phosphate (P_i_) by *CANT1*. Thanks to UDP hydrolysis, glycosyltransferase reactions are not inhibited by the product (negative feedback) and UMP is exchanged with cytosolic UDP sugars through an antiporter exchanger.

It has been suggested that *CANT1* encodes a uridin diphosphate (UDP) nucleotidase putatively needed for proteoglycan synthesis and involved in vesicular trafficking in Golgi apparatus by calcium release through inositol 1,4,5-triphosphate receptor activation [Huber et al., 2009]. Our results support the hypothesis that *CANT1* in the ER/Golgi compartment might play a role in proteoglycan synthesis through the hydrolysis of UDP a product of glycosyl transferase reactions. Thanks to UDP removal, glycosyltransferase reactions are not inhibited and uridin monophosphate (UMP) is exchanged with cytosolic UDP sugars through an antiporter exchanger (Fig. 5). Thus, functional impairment of *CANT1* would result in increased Golgi UDP level causing feedback inhibition of glycosyltranferase activities and reduced transport of UDP sugars in the Golgi compartment, affecting, overall, GAG synthesis. In this condition, reduced GAG synthesis occurs when cells are pressed to synthesize high amount of proteoglycans as is the case during incubation with β-d-xyloside in vitro or, in vivo, in tissues with high proteoglycan content (i.e., cartilage). Interestingly, reduced hydrodynamic size of GAG chains was also detected, suggesting a reduced elongation rate of GAG chains, even if enhanced oligosaccharide degradation might not be excluded. Finally, our finding of normal hyaluronic acid synthesis, which occurs on the plasma membrane through hyaluronan synthases [Vigetti et al., 2009] further support the involvement of *CANT1* in the ER/Golgi compartment

We conclude that *CANT1* is the major gene responsible for DD and expand the spectrum of hand anomalies observed in this disorder. We also demonstrated the role of CANT1 in the rate of proteoglycan synthesis. Ongoing studies will hopefully lead to identify other disease gene(s) responsible for DD type 2 presumably involved in proteoglycan synthesis.
