# Supplementary material for: Further Delineation of CANT1 Phenotypic Spectrum and Demonstration of Its Role in Proteoglycan Synthesis
Source: Hum Mutat. 2012 Apr 26;33(8):1261–6. doi: 10.1002/humu.22104 (PMC3427906; doi:10.1002/humu.22104)
Supplement: Supplementary file 1 [file humu0033-1261-SD1.pdf]

Supp. Table S1. Sequences of *CANT1* primers

**Primer name**	**Sequence (5’-3’)**
CANT1-1 F	AAAAACACAAACCACAGCTC
CANT1-1 R	GAGTCAGGGGGCAGAGAT
CANT1-2(1) F	TGTCTATGATCCTATCCCACTTC
CANT1-2(1) R	GAGTGCATAGACTCATTCCATTC
CANT1-2(2) F	GAACTAAGATGTGACTGGGCTTG
CANT1-2(2) R	AATCAGGTCGGATAGCTCCAT
CANT1-2(3) F	AGAGGAAAACACCTGGTTCAGTT
CANT1-2(3) R	ATAAGCTCTTCCTCCTAGAAACT
CANT1-3 F	AGTCAGGTGCGGGGTCTAAG
CANT1-3 R	GGCTGTGGAAGCCACACT
CANT1-4(1) F	AGCCCCTTGCCTGCTACT
CANT1-4(1) R	AGCATGGAAAGCAGTTCAGAC
CANT1-4(2) F	GGTTGGAGGTCTGGACAGG
CANT1-4(2) R	GTGATAAGATTTGGTGATTCCAC
CANT1-4(3) F	GGCTGTGCAGTACTTTGAAGTTT
CANT1-4(3) R	GCACAGGGACAGCCAGAG

Supp. Table S2. Sequences of *CHST3* primers

**Primer name**	**Sequence (5'-3')**
CHST3-1 F	CTCCCCTCTCTCCCCTCTCT
CHST3-1 R	CGGTGTGGGTGTGCGCAGAT
CHST3-2 F	ACATCCTCTGCATTCCTCGT
CHST3-2 R	CATTTGCAGGTCCACAGAGA
CHST3-3(1) F	TGGAGGACTGCTTAGGGTTG
CHST3-3(1) R	CCCTGCTGGTTGAAGAACTC
CHST3-3(2) F	CAGGGGAGGAAGAGGAAGAG
CHST3-3(2) R	GCGGTTCTTGCAGTGGTACT
CHST3-3(3) F	CACCTGACTCAGTTCATGTTCC
CHST3-3(3) R	GTAGCGCACCAGCATGTAGC
CHST3-3(4) F	GAGGGAAGAGGAGGTGCAG
CHST3-3(4) R	TTCACGAGGAGGGGCATAC
